# Macrophages CD163+ and Factor XIIIa+ Provide a First-Line Defence against Proliferative Verrucous Leukoplakia Antigens

**DOI:** 10.3390/ijms24065242

**Published:** 2023-03-09

**Authors:** Mariana Paravani Palaçon, Camila de Oliveira Barbeiro, Darcy Fernandes, Mariel Ruivo Biancardi, Heitor Albergoni Silveira, Túlio Morandin Ferrisse, Jorge Esquiche León, Omar Kujan, Andreia Bufalino

**Affiliations:** 1 Oral Medicine, Department of Diagnosis and Surgery, School of Dentistry, São Paulo State University (Unesp), Araraquara 14801-903, SP, Brazil; 2 Oral Pathology, Department of Stomatology, Public Oral and Forensic Dentistry, Ribeirão Preto Dental School, University of São Paulo (FORP/USP), Ribeirão Preto 14040-904, SP, Brazil; 3 UWA Dental School, The University of Western Australia, Perth, WA 6009, Australia

**Keywords:** proliferative verrucous leukoplakia, macrophages, dendritic cells, malignant transformation, oral

## Abstract

This study aimed to evaluate the density of the dendritic cells (DCs) and macrophages in oral leukoplakia (OL) and proliferative verrucous leukoplakia (PVL) by immunohistochemical analysis. We analysed paraffined tissue samples of PVL (*n* = 27), OL (*n* = 20), and inflammatory fibrous hyperplasia (*n* = 20) as the control group using the immunomarkers for DCs (CD1a, CD207, CD83, CD208 and CD123) and macrophages (CD68, CD163, FXIIIa and CD209). A quantitative analysis of positive cells in the epithelial and subepithelial areas was determined. Our results showed a reduction in CD208+ cells in the subepithelial area of the OL and PVL compared to the control. Additionally, we found a higher density of FXIIIa+ and CD163+ cells in the subepithelial area in PVL compared to the OL and control. Four-way MANOVA revealed a relationship between increased CD123+ cell density in the subepithelial area of “high-risk” samples regardless of disease. Macrophages provide the first line of defence against PVL antigens, suggesting a distinct pattern of innate immune system activation in PVL compared to OL, which may contribute to the complexity and the high rate of malignant transformation in the PVL.

## 1. Introduction

Proliferative verrucous leukoplakia (PVL) is an oral potentially malignant disorder (OPMD) which has a high rate of malignant transformation (>40%) and resistance to all treatment modalities [1]. PVL was previously regarded as an aggressive variant of oral leukoplakia (OL). Nevertheless, unlike OL, PVL appears as recalcitrant non-homogenous leukoplakia affecting multiple sites with progressive involvement of contiguous or non-contiguous areas over time [2]. Further, PVL is more common in non-smoking elderly women and does not seem to be associated with well-known risk factors such as tobacco use, alcohol consumption and areca nut chewing [1,3]. Hence, these clinical aspects associated with aggressive biological behaviour allow PVL to be classified as distinct from OL [1,2]. The advanced understanding of the biological processes that may be damaged in PVL, including the mechanisms related to the immune system, could help develop more efficient therapeutic modalities for this OPMD that lacks proper therapy. 

Recently, a study revealed that lesions of patients diagnosed with PVL showed an increase in CD8+ T cells compared to patients diagnosed with OL [4]. However, the absence of elevated levels of cytokines commonly related to activated CD8+ T cells in PVL suggests that these cells are driven to anergy/exhaustion [4]. With that, it is well-recognized that antigen-presenting cells (APCs), such as dendritic cells (DCs) and macrophages, are crucial to T cell activation [5,6]. The current evidence supports a relationship between DCs and better prognosis in patients affected by oral squamous cell carcinoma (OSCC) and oral epithelial dysplasia (OED) has been previously reported [7,8]. Another study showed that Langerhans cells (DCs expressing CD1a) and CD8+ T cells are more abundant in dysplastic OL than non-dysplastic OL [9]. Expectedly, the number of Langerhans cells and CD8+ T cells are increased in OSCC compared to OL [9]. Furthermore, a positive correlation between the intraepithelial CD4+ T cell rate and the CD163+ macrophage population was found in mild and moderate OED lesions [10]. In addition, the Th1 profile (CXCR3+, CCR5+ and IFN-induced gene products) was observed in the same lesions, whereas the Th2 phenotype was scarce in these lesions, demonstrating that CD163+ macrophages in OL appear to possess an M1 phenotype characterised by the expression of IFN-induced gene products and coexpression of CD163 and STAT1 [10]. A negative correlation between several CD8+ T cells and CD163+ macrophages in OL seems to support the hypothesis that high infiltration of CD8+ T cells is associated with an M1 phenotype and improves clinical results [11]. It should be noted that PVL patients were not evaluated in these studies, and the presence of APCs needs to be characterised to advance the understanding of their role in the malignant transformation of this OPMD. Further, the participation of immune cells and immunological mechanisms related to the malignant transformation of OPMDs, particularly in PVL, are poorly understood. In addition, studies show that macrophages and DCs have an overlapping expression of several markers derived from monocytes and probably have similar functions [12,13]. Therefore, this study investigates the density and correlation of DCs (CD1a, CD207, CD83, CD208 and CD123) and macrophages (CD163, CD68, CD209, factor XIIIa) cells by immunohistochemistry in OL and PVL to understand the role of these immune cells in these lesions which share some clinicopathological features but present distinct clinical behaviour.

## 2. Results

### 2.1. Clinicopathological Features of OL and PVL Patients

Our analysis was performed using paraffin blocks from 20 patients with the previous diagnosis with OL and 27 paraffin blocks obtained at different times of the clinical follow-up of 12 patients diagnosed with PVL. The 27 paraffin blocks belonging to the PVL group were obtained over an average follow-up period of 88 months with an average of 2 biopsies per individual. In this analysis, only samples presenting microscopical aspects ranging from hyperkeratosis without or varying degrees of dysplasia were included. The epidemiological profile of the patients included in this study revealed that most individuals in the OL group were male (*n* = 13; 65%), smokers (13 patients; 65%), with a mean age of 53.3 years (30 to 79 years). In addition, all individuals in the OL group had a single lesion, with the vast majority showing a lesion with a total size of less than 3 cm (*n* = 13; 90%). All patients with OL underwent a single biopsy, most of which were classified as “low-risk” (*n* = 13; 65%). Analysing the clinical–pathological characteristics of the PVL group, we observed that half patients were female (*n* = 6; 50.0%), smokers (four patients; 33.3%,) and with a mean age of 64.3 years (41 to 79 years old). The average number of biopsies of patients with PVL was 2.3, with a total of 27 samples. Most PVL samples were “low-risk” (*n* = 16; 59.3%). The malignant transformation rate in our population was 15.0% for OL and 58.3% for PVL. The recurrence rates were 0% and 75.0% for OL and PVL, respectively. All the clinicopathological data from the participants are presented in Table 1.

### 2.2. Assessment of Density and Distribution of Dendritic Cells and Macrophages

For the characterisation of dendritic cells, the markers CD1a, CD207, CD83, CD208 and CD123 with histomorphological analysis were used to assess their density and distribution in the OL and PVL. Cells with dendritic morphology were found in the intraepithelial and subepithelial areas. CD1a+ and CD207+ cells were more present in the intraepithelial area, whereas CD83+ and CD208+ positive cells were found most frequently in the subepithelial area in all groups. On the other hand, CD123+ positive cells showed a homogeneous distribution in both compartments. We found an overall reduction in DC markers in OL and PVL compared with the control group, except for the CD83+ cells that showed a similar density between groups. A statistically significant reduction in CD208+ cells in the subepithelial area of the OL (*p* = 0.001; 95% CI = 1.13–5.01) and PVL (*p* = 0.001; 95% CI = 1.19–4.95) compared to the control was noticed. Although no relevant statistical differences were found, a clear reduction of a subpopulation of CD1a+ and CD207+ cells was found in OL and PVL compared to control in the intraepithelial and subepithelial areas. 

**Figure 1:**
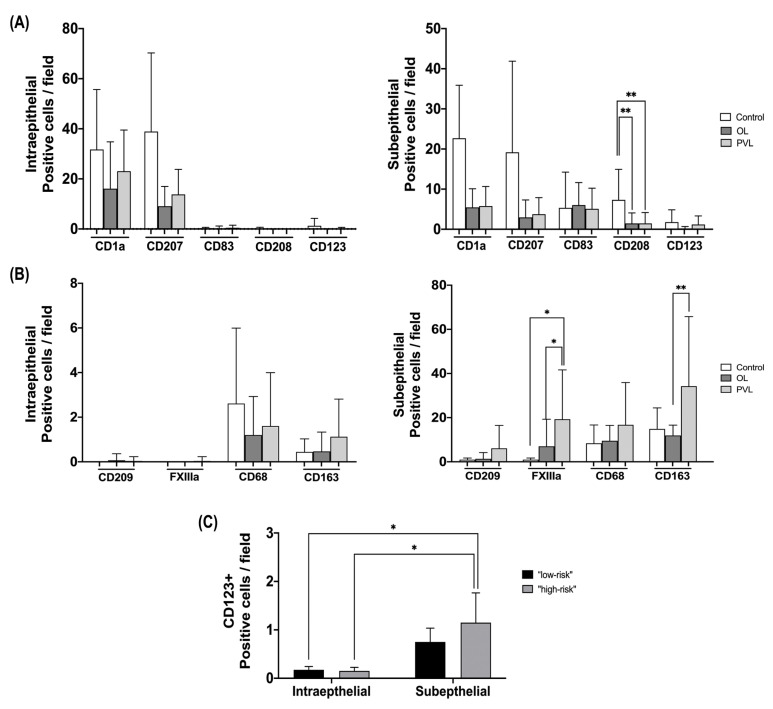
Results of immunostaining quantification and statistical differences for DC and macrophage markers. (**A**) CD1a+, CD207+, CD83+, CD208+ and CD123+ cells with dendritic morphology were counted in the intraepithelial and subepithelial areas. (**B**) CD209+, FXIIIa+, CD68+ and CD163+ macrophages were counted in the intraepithelial and subepithelial areas. (**C**) Four-way MANOVA analysis shows the differences between the increase in the density of CD123+ cells in the subepithelial area of “high-risk” to malignant transformation samples compared to the density of these cells in the intraepithelial area of “low-risk” and “high-risk” to malignant transformation samples, regardless of disease. * *p* < 0.05 and ** *p* < 0.01. OL = oral leukoplakia; PVL = proliferative verrucous leukoplakia.

**Figure 2:**
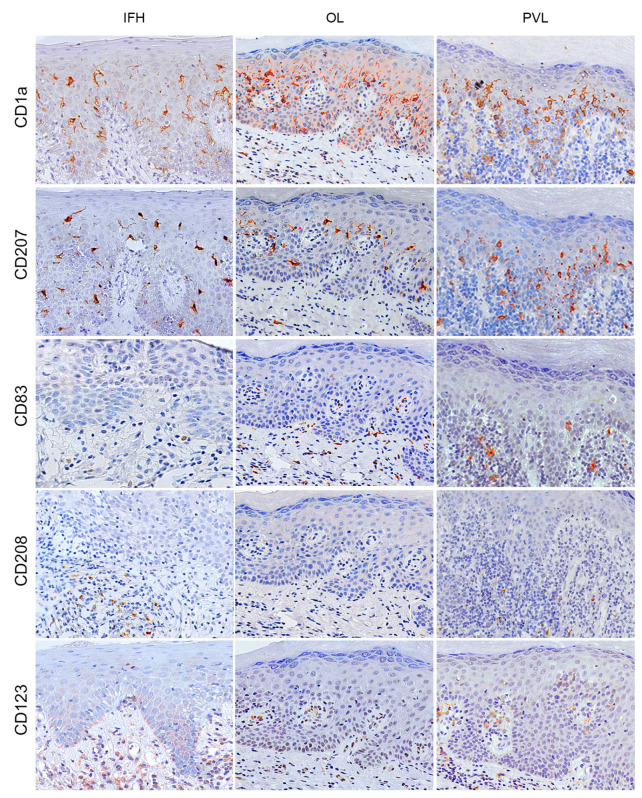
Representative image of the immunological staining pattern for different DC markers by the immunohistochemical technique. The columns show the study groups, the first column being the control group (IHF), the second column the OL group and the third column the PVL group. The lines show the markers that characterized DC for different groups, including CD1a, CD207, CD83, CD208 and CD123. Positive cells are represented by brown staining, and counterstained was performed using Carazzi’s hematoxylin. Original magnification: ×200 for all images. DC = dendritic cells; IHF = inflammatory fibrous hyperplasia; OL = oral leukoplakia; PVL = proliferative verrucous leukoplakia.

**Figure 3:**
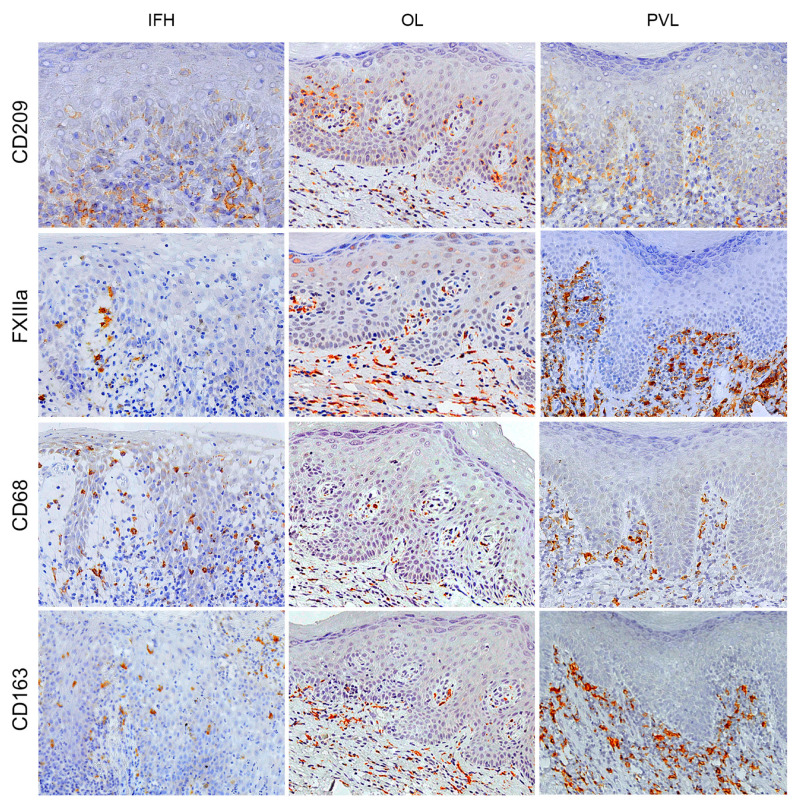
Representative image of the immunological staining pattern for different DC markers by the immunohistochemical technique. The columns show the study groups, the first column being the control group (IHF), the second column the OL group and the third column the PVL group. The lines show the markers used to characterized macrophages for different groups, including CD209, FXIIIs, CD68, and CD163. Positive cells are represented by brown staining, and counterstained was performed using Carazzi’s hematoxylin. Original magnification: ×200 for all images. DC = dendritic cells; IHF = inflammatory fibrous hyperplasia; OL = oral leukoplakia; PVL = proliferative verrucous leukoplakia.

In this study, we used CD68, CD163, FXIIIa, and CD209 immunostaining to characterise the macrophage population. Generally, a higher macrophage density was found in all groups’ subepithelial areas. Furthermore, this population showed a higher mean density in both OPMDs than the control, different from observed DC markers. Multivariate analysis revealed no statistical difference in the intraepithelial area, but we found a significant difference in the subepithelial density of FXIIIa+ and CD163+ cells. A higher density of FXIIIa+ cells was found in the subepithelial area of the PVL compared to the OL (*p* = 0.03; 95% CI = 0.36–13.07) and control groups (*p* = 0.04; 95% CI = 0.09–19.40). In addition, an increase in macrophages CD163+ density in the PVL compared with OL (*p* = 0.001; 95% CI = 4.34–20.67). The immunostaining quantification and statistical differences for DC and macrophage markers may be observed in Figure 1 and all the immunohistochemical staining patterns for the primary antibodies are presented in Figure 2 and Figure 3.

### 2.3. Multivariate Analysis of Variance with Four Independent Factors

In our study, four independent variables were considered, including clinicopathological diagnosis (OL, PVL and IFH), histological areas (intraepithelial and subepithelial areas), binary system of grading oral epithelial dysplasia (“low-risk” and “high-risk” lesions) and malignant transformation (no history of malignant transformation and malignant transformation to squamous cell carcinoma, and verrucous carcinoma). The dependent variables were the density of DC and macrophages positive cells to the markers included in this study. Thus, the study design required using a four-way multivariate analysis of variance. 

In this analysis, we found only a statistical significance to the biomarker CD123 in relation to histological areas and grading of oral epithelial dysplasia independent variables. The post hoc test of Tukey was performed and showed a significant increase in the density of CD123+ cell in the subepithelial area of “high-risk” samples compared to the density of these cells in the intraepithelial area of “low-risk” (*p* = 0.019; 95% CI = 0.11–1.82) and “high-risk” (*p* = 0.048; 95% CI = 0.005–1.98) samples, regardless of disease (Figure 1). 

### 2.4. Dendritic Cells and Macrophages Markers Correlation in OL and PVL

Pearson’s correlation coefficient was applied to evaluate the association between DCs and macrophage markers in the OL and PVL (Table 2). Interestingly, most correlations were found in the PVL group, classified as a positive weak/moderate association. Overall, all markers for both DCs and macrophages correlated with each other in PVL. On the other hand, there was only a statistically significant correlation between CD68 with CD83 (*p* = 0.0058; r = 0.5933; R^2^ = 0.352) and CD208 (*p* = 0.03; r = 0.4830; R^2^ = 0.2333) in the OL. Thus, we suggest that different DCs and macrophage subtypes have a weak effect on the density of each cell population. The multiple correlations in PVL may indicate the activation of several distinct immune pathways in this OPMD.

## 3. Discussion

DCs and macrophages are the first lines of immune surveillance; both cell types can detect pathogens or tissue damage and perform initiation and/or regulation of adaptative immunity through antigen presentation to T cells [14]. 

Several studies have addressed the importance of DCs for the prognosis in patients affected by different cancers [7,15,16,17]. The DCs present antigens to CD4+ and CD8+ T cells in the lymph nodes, and as the DCs act at the interface of the innate and adaptive immune systems, they may be used as targets in cancer immunotherapy [18]. These immune cells may be classified into subsets based on an integrated approach considering surface phenotype, expression of unique and conserved molecules, ontogeny, and function. In this sense, immature DC, characterised by expressing molecules such as CD1a and CD207, recognise capture antigens and produces chemokines acquiring a mature DC phenotype [19]. This mature cell migrates to the lymph node to present antigens to naïve T cells and express MHC class II and co-stimulatory molecules such as CD83 and CD208 [19,20]. 

This study demonstrated a general decrease of DCs population in both OPMDs compared to the control group. Nevertheless, it should be noted that previously published studies used samples of healthy oral mucosa as a control group. Souto et al. (2018) found an increase in CD83+ cells in the large (>10 mm) and dysplastic lesions of OL but could not find any relationship with CD1a+ count. Indeed, some groups reported a significant relationship between high-grade dysplasia and a decrease in CD1a+ Langerhans cell density [7,21], whereas others have observed an increase in CD1a+ cells in dysplastic tissue [22]. In another study, no difference in the CD1a+ frequency was observed when comparing OL samples with and without dysplasia [9]. 

Additionally, we found a statistically significant depletion of CD208+ cells in the subepithelial area in both OPMDs compared to the control group. A previous study analysing OSCC samples found that the number of CD208+ cells is higher in the tumour stroma and the tumour nest compared to the non-neoplastic tissue [23]. In addition, they found that only the number of CD208+ in the tumour stroma was associated with lymph node positivity in OSCC patients; despite this, it did not correlate with the overall survival of patients [23]. Pellicioli et al. (2017) found a decrease in the CD83+ cells (mature DCs) and an increase in the CD303+ cells (plasmacytoid DC subtype) in OSCC compared to the OED group [24]. Our analysis found a relationship between an increase in CD123+ cell density (plasmacytoid DC subtype) in the subepithelial area of “high-risk” samples regardless of disease but not with malignant transformation. This finding may explain why the current grading system of OED has little value in predicting malignant transformation, especially in PVL. 

Our results also showed an increase in the number of macrophages in PVL compared to OL and control groups. In this sense, it has been reported that there are two different activation states of macrophages called M1 and M2. Classically activated macrophages expressing CD68 (M1) are correlated to antigen presentation and inflammatory response, whereas alternatively activated macrophages expressing CD163 (M2) may be involved in cancer growth, angiogenesis, metastasis and therapy resistance [25,26]. We found an increased number of CD163+ cells in PVL compared to OL, suggesting M2 polarisation in PVL. A recent study found infiltrating macrophages with M2 polarisation in OL with malignant transformation compared to OL without malignant transformation [27]. In this same study, the authors showed that OSCC samples derivatives of OL patients with malignant transformation had the highest macrophage infiltration and strongest M2 polarisation, concluding that increased macrophage infiltration and M2 polarisation was associated with the development of oral cancer in OL [27]. An additional study showed a prognostic significance between CD68+ and CD163+ in head and neck squamous cell carcinoma, and they found that CD68+ has no prognosis in patients, whereas CD163+ predicts poor prognosis [28]. 

Although factor XIIIa has been used as a DC marker, distinct from Langerhans cells, FXIIIa+ cells share some features common to monocyte and macrophages [29,30,31,32]. In a previous study, it was observed that double labelling with antibodies against CD11c and FXIIIa showed two distinct cell populations, unlike double labelling with CD68+, which partially coexpress factor XIIIa [12]. So, they concluded that factor XIIIa is not a skin disease-specific marker and indicates a possible common mechanism of macrophage activation in dermatological diseases [12]. Similarly, another study showed that a major subset of dendritic-appearing macrophages dermal cells CD209+ coexpress molecules as CD68, CD14, and CD163. These cells appear to comprise a novel part of the innate immune response in the resident skin immune system [13,33]. It should be noted that these previous studies showed cells co-expressing CD209 and FXIIIa with classic macrophage markers (CD68 and CD163) by double immunofluorescence labelling. Considering these facts, in this study factor XIIIa and CD209 were used to characterise macrophages, and it was noticed that the PVL presents a high density of FXIIIa compared to OL and control. However, using single immunohistochemical labelling in our study may be a limitation, as well as the vast majority of studies that include OPMDs.

Finally, in the present study, DCs and macrophage markers showed a weak correlation, indicating that when one variable increases, the other variable tends to increase as well, but in a weak or unreliable manner. Thus, the large number of weak correlations between cellular subtypes and the higher density of macrophages observed in PVL compared to OL may suggest that macrophages of the innate immune system provide a first line or main line of defence against PVL antigens. In this context, exhausted CD8+ T cells in cancer express factors that actively recruit monocytes and shape their differentiation. Still, these cells engage in interactions that fail to activate T cells and prime them for exhaustion [34]. Therefore, the results found in our study favour the previous hypothesis that CD8+ T-cell infiltrates in the PVL are in a state of exhaustion [4]. The malignant transformation of oral epithelial disorders is a complicated process where immunomodulation events may play a role in evading the immune system and contributing to the potential cancer progression in these lesions [35].

## 4. Materials and Methods

### 4.1. Study Population

This study was conducted following the Declaration of Helsinki after receiving approval from the local ethics committee (CAAE: 34361814.9.0000.5416). All participants agreed to participate in the study and provided written informed consent. In total, 68 oral tissue specimens were included in this study, with 20 inflammatory fibrous hyperplasias (IFH) as the control group, 20 samples of OL and 27 samples of PVL. OL and PVL cases were included if they met the diagnostic criteria of OL and PVL as outlined by the 2021 WHO classification of OPMDs [2]. Briefly, a case is considered OL if it was a predominantly white lesion that cannot be rubbed off after excluding other clinically recognizable white or white/red lesions as outlined by Warnakulasuriya et al. (2021). Likewise, PVL is considered when the patient presented with multiple, thick, white patches in more than two different oral sites, with recognizable surface change. Cases were excluded if the samples showed microscopically squamous cell carcinoma or verrucous carcinoma or showing. The IFH was selected as a control group because it is a reactive lesion with chronic inflammation that microscopically shows hyperkeratotic epithelium, containing areas of acanthosis alternating with atrophic areas, and it has no risk of malignant transformation. All patients had a minimum follow-up of five years after confirmation of the diagnosis. The PVL archive had two or three biopsies from each patient collected from different oral sites. All included cases received a histopathological evaluation using hematoxylin–eosin-stained sections by two pathologists independently, and any discrepancies were resolved by discussion with a third pathologist to arrive at a consensus on the final diagnosis with emphasis on the presence and grade of epithelial dysplasia. The cases were classified into “low risk” and “high risk” lesions using the binary grading system of OED [36]. OL final diagnosis was established for cases that clinically showed a simple leukoplakia and PVL final diagnosis clinically showed multiple leukoplakias varying from flat white to verrucous plaques, which showed spreading over time. 

### 4.2. Immunohistochemical Analysis

We obtained consecutive (serial) cuts of 3-μm in the paraffined tissue block for the immunohistochemical analysis. The antigens were retrieved after deparaffinisation and rehydration in ethanol solutions. Afterwards, we blocked endogenous peroxidase activity with 0.1% H2O2 for 5 min at room temperature. The slides were incubated separately with primary antibodies against CD1a (Dako, Carpinteria, CA, USA), CD83, CD123, Factor XIIIa, CD163 (Leica Biosystems, Wetzlar, Germany), CD207 (Monosan, Uden, The Netherlands), CD209, CD68 (DakoCytomation, Glostrup, Denmark), and CD208 (Dendritics, Lyon, France) followed by the LSAB method (Dako, Carpinteria, CA, USA) and the reactions were developed by incubating the sections with 0.6 mg/ml 3,3’diaminobenzidine tetrahydrochloride (Sigma-Aldrich, Saint Louis, MO, USA). The slides were counterstained with Carazzi’s hematoxylin. Negative controls were obtained by omitting primary antibodies. The results were observed under an optical microscope with 200× magnification (Leica Biosystems, Wetzlar, Germany). Ten consecutive and representative fields of the intraepithelial and subepithelial areas were selected for photomicrography (Leica Application Suite–LAS). Two independent examiners counted positive cells using the Image J software (version 1.52, NIH, Bethesda, Rockville, MD, USA). 

### 4.3. Statistical Analysis

Descriptive statistics were used to evaluate data from the patient population and immunohistochemical results. To evaluate the normality distribution of the data, we used the Shapiro–Wilk test and the following descriptive aspects: asymmetry and kurtosis. The presence of outliers was checked. Box’s test of equality of covariance matrices was utilised to verify the homoscedasticity, and Levene’s test of equality variances was used. The multivariate analysis of variance (MANOVA) with two and four independent factors was performed, two-way and four-way MANOVA, respectively. A Tukey post hoc test was chosen to evaluate the differences between the groups. The effect factor was calculated when p value was statistically significant. Additionally, was performed a correlation study and a simple linear regression with the markers for OL and PVL. A *p*-value of < 0.05 was regarded to be statistically significant. Statistical analysis was performed by IBM SPSS Statistics 20.0 (IBM, Armonk, NY, USA).

## 5. Conclusions

In conclusion, we noticed a distinct pattern of innate immune system activation in PVL compared to OL, which may contribute to the complexity and the high rate of malignant transformation in the PVL.

## Figures and Tables

**Table 1:** Clinicopathological characteristics of enrolled patients in our study and diagnosed with OL and PVL.

Clinicopathological features	OL (*n* = 20)	PVL (*n* = 12)
	*n* (%)	*n* (%)
**Average age (years)**	53.3	64.3
**Gender**		
Male	13 (65.0)	6 (50.0)
Female	7 (35.0)	6 (50.0)
**Ethnicity**		
White	17 (85.0)	12 (100)
Nonwhite	3 (15.0)	0 (0.0)
**Tobacco**		
No	7 (35.0)	8 (66.7)
Yes	13 (65.0)	4 (33.3)
**Alcohol**		
No	20 (86.9)	10 (83.3)
Yes	3 (13.1)	2 (16.7)
**Oral sites** ^++^		
Right buccal mucosa	4 (20.0)	6 (50.0)
Left buccal mucosa	4 (20.0)	6 (50.0)
Floor of the mouth	0 (0.0)	4 (33.3)
Maxillary gingivae	0 (0.0)	2 (16.7)
Mandibular gingivae	2 (10.0)	4 (33.3)
Upper labial mucosa	0 (0.0)	2 (16.7)
Lower labial mucosa	1 (5.0)	2 (16.7)
Tongue	7 (35.0)	5 (41.7)
Palate	2 (10.0)	3 (25.0)
**Binary system of dysplasia grade** ^++^		
Low risk	13 (65.0)	16 (59.3)
High risk	7 (35.0)	11 (40.7)
**Malignant transformation**		
No	17 (85.0)	5 (41.7)
Yes	3 (15.0)	7 (58.3)
**Recurrence**		
No	20 (100)	3 (25.0)
Yes	0 (0.0)	9 (75.0)

^++^ The 27 paraffin blocks belonging to the PVL group were obtained from 12 patients with an average of two biopsies per individual during a clinical follow-up period of approximately 88 months. OL = oral leukoplakia; PVL = proliferative verrucous leukoplakia.

**Table 2:** Results of Pearson’s correlation between DCs and macrophages to different study groups.

		OL			PVL		
Markers		*p*-Value	r	R^2^	*p*-Value	r	R^2^
CD1a	CD209	0.4052	−0.1969	0.0388	0.6081	0.1033	0.0107
CD1a	FXIIIa	0.0844	0.3954	0.1563	**0.0494**	0.3817	0.1457
CD1a	CD68	0.0891	0.3900	0.1521	0.1009	0.3224	0.1039
CD1a	CD163	0.0863	0.3951	0.1561	0.1238	0.3034	0.0921
CD207	CD209	0.4395	−0.1832	0.0335	0.5202	0.1294	0.0167
CD207	FXIIIa	0.7874	0.0644	0.0041	0.2285	0.2397	0.0574
CD207	CD68	0.5933	0.1271	0.0162	**0.0108**	0.4825	0.2328
CD207	CD163	0.4042	0.1974	0.039	0.0784	0.3445	0.1187
CD83	CD209	0.7969	0.0615	0.0038	0.0792	0.3436	0.1181
CD83	FXIIIa	0.1267	0.3531	0.1247	**0.0199**	0.4452	0.1982
CD83	CD68	**0.0058**	0.5933	0.352	**<0.0001**	0.6852	0.4695
CD83	CD163	0.2093	0.2934	0.0861	**0.0010**	0.5958	0.3550
CD208	CD209	0.3515	0.2199	0.0484	0.0867	0.3359	0.1128
CD208	FXIIIa	0.8750	0.0376	0.0014	**0.0055**	0.5195	0.2699
CD208	CD68	**0.0309**	0.4830	0.2333	**0.0046**	0.5284	0.2793
CD208	CD163	0.2979	0.2449	0.0600	**0.0318**	0.4139	0.1713
CD123	CD209	0.4605	−0.175	0.0306	**0.0087**	0.4948	0.2448
CD123	FXIIIa	0.9569	−0.0129	0.0002	0.5526	−0.1195	0.0143
CD123	CD68	0.9750	−0.0075	0.0001	0.5364	0.1244	0.0155
CD123	CD163	0.5894	0.1284	0.0165	0.8455	0.0393	0.0015

*p*-Value < 0.05 was considered as statistical significance. The values in bold in the table were to highlight the results that showed a statistically significant difference. DC = dendritc cells; OL = oral leukoplakia; PVL = proliferative verrucous leukoplakia; r = Person correlation coefficient; R2 = coefficient of determination.

## Data Availability

Anonymised source data are available upon reasonable request.
